# Genetic Susceptibility to Enteric Fever in Experimentally Challenged Human Volunteers

**DOI:** 10.1128/iai.00389-21

**Published:** 2022-03-07

**Authors:** Amber Barton, Jennifer Hill, Sagida Bibi, Liye Chen, Claire Jones, Elizabeth Jones, Susana Camara, Sonu Shrestha, Celina Jin, Malick M. Gibani, Hazel Dobinson, Claire Waddington, Thomas C. Darton, Christoph J. Blohmke, Andrew J. Pollard

**Affiliations:** a Oxford Vaccine Group, Department of Paediatrics, University of Oxford and the NIHR Oxford Biomedical Research Centre, Oxford, United Kingdom; b Clinical Research Department, London School of Hygiene and Tropical Medicine, London, United Kingdom; c Nuffield Department of Orthopaedics, Rheumatology and Musculoskeletal Sciences, University of Oxford, Oxford, United Kingdom; d Department of Infectious Disease, Imperial College London, London, United Kingdom; e Department of Medicine, University of Cambridge, Cambridge, United Kingdom; f Department of Infection, Immunity and Cardiovascular Disease, University of Sheffield, Sheffield, United Kingdom; g Florey Institute for Host-Pathogen Interactions, University of Sheffield, Sheffield, United Kingdom; University of California San Diego School of Medicine

**Keywords:** typhoid fever, single nucleotide polymorphism, transcriptome, *Salmonella* Typhi, genomics, unfolded protein response, HLA antigens

## Abstract

Infections with Salmonella enterica serovars Typhi and Paratyphi A cause an estimated 14 million cases of enteric fever annually. Here, the controlled nature of challenge studies is exploited to identify genetic variants associated with enteric fever susceptibility. Human challenge participants were genotyped by Illumina OmniExpress-24 BeadChip array (*n* = 176) and/or transcriptionally profiled by RNA sequencing (*n* = 174). While the study was underpowered to detect any single nucleotide polymorphisms (SNPs) significant at the whole-genome level, two SNPs within *CAPN14* and *MIATNB* were identified with *P* < 10^−5^ for association with development of symptoms or bacteremia following oral S. Typhi or *S.* Paratyphi A challenge. Imputation of classical human leukocyte antigen (HLA) types from genomic and transcriptomic data identified HLA-B*27:05, previously associated with nontyphoidal Salmonella-induced reactive arthritis, as the HLA type most strongly associated with enteric fever susceptibility (*P* = 0.011). Gene sets relating to the unfolded protein response/heat shock and endoplasmic reticulum-associated protein degradation were overrepresented in HLA-B*27:05^+^ participants following challenge. Furthermore, intracellular replication of S. Typhi is higher in C1R cells transfected with HLA-B*27:05 (*P* = 0.02). These data suggest that activation of the unfolded protein response by HLA-B*27:05 misfolding may create an intracellular environment conducive to S. Typhi replication, increasing susceptibility to enteric fever.

## INTRODUCTION

Salmonella enterica serovars Typhi and Paratyphi A cause an estimated 14 million cases of enteric fever per year, resulting in 135,000 deaths (1). Several risk factors have been identified for enteric fever, including poor sanitation and flooding (2). Individual host factors also likely contribute to disease susceptibility. Human challenge models, where volunteers are deliberately exposed to a pathogen, have been developed to study the biology of enteric fever and test experimental vaccines. Despite ingesting the same inoculation dose of bacteria, some challenged individuals remain infection free, while others develop bacteremia or symptoms consistent with enteric fever (3, 4). This could be explained in part by unmeasured factors such as effective bacterial dose reaching the intestinal mucosa, or other random effects not amenable to control. Alternatively, certain participants may have an innate resistance or susceptibility to enteric fever: in unvaccinated human challenge participants undergoing homologous rechallenge with S. Typhi, those who did not develop enteric fever on the first exposure were less likely to develop enteric fever on the second exposure (5). Host genetics could play a role in this resistance. Genome-wide association studies (GWAS) are frequently used to find associations between genetic variants and complex non-Mendelian traits, with the aim of identifying genes which may provide insight into the pathology of a disease. For example, a GWAS identified polymorphisms in the NOD2 pathway as being associated with leprosy susceptibility (6). NOD2 activation was later found to induce dendritic cell differentiation, which may protect against disease progression (7). In the case of Salmonella infections, GWAS have revealed the human leukocyte antigen (HLA)-DRB1*04:05 allele as conferring resistance against typhoid fever (8) and a locus in *STAT4* as being associated with nontyphoidal Salmonella bacteremia (9).

In epidemiological studies, genetic heterogeneity in the pathogen is a confounder to the infected human host’s individual susceptibility to that pathogen, as illustrated by studies of tuberculosis, in which host single nucleotide polymorphisms (SNPs) predispose individuals to infection with a particular strain only (10). In studies performed at our center to date, only three strains of Salmonella have been used as a challenge agent, which has allowed us to statistically control for pathogen heterogeneity. All participants are exposed to the pathogen under highly controlled conditions, whereas in the field “noninfected controls” may have avoided infection due to lack of environmental exposure rather than having been exposed and resisted infection. Furthermore, prior exposure modifies susceptibility to enteric fever (5), which is difficult to account for in the field as Salmonella exposure is likely frequent during childhood in settings of endemicity. However, this can be managed in challenge studies through strict inclusion criteria and careful screening, including exclusion of participants who had received a typhoid vaccine or lived in an area of typhoid endemicity. Despite the advantages of human challenge studies, to our knowledge a GWAS has not previously been carried out on human challenge participants. Here, we exploit this unique setting and investigate how differences in host genetics relate to outcome of challenge. We identify SNPs within the genes *CAPN14* and *MIATNB* as having *P* < 10^−5^ for association with development of enteric fever symptoms or bacteremia following exposure. We find that HLA-B*27:05 is the HLA type most strongly associated with enteric fever susceptibility, enhancing intracellular replication of S. Typhi.

(This work was previously presented at the British Society for Immunology [BSI] virtual conference, December 2020, United Kingdom, abstract ID 989.)

## RESULTS

### No SNPs were significantly associated with the outcome of challenge at the genome-wide level.

**FIG 1:**
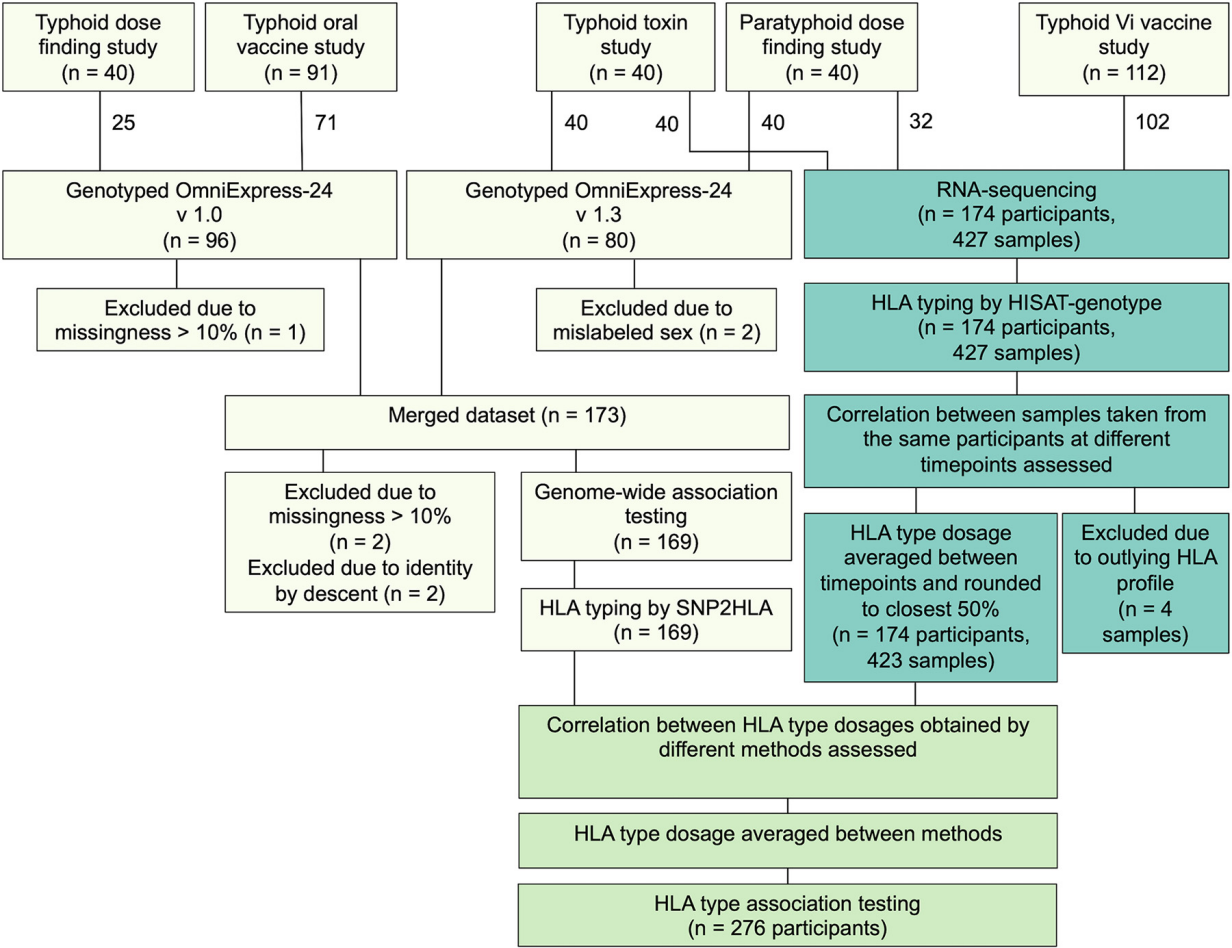
Number of participants and samples at each stage of the analysis pipeline.

**FIG 2:**
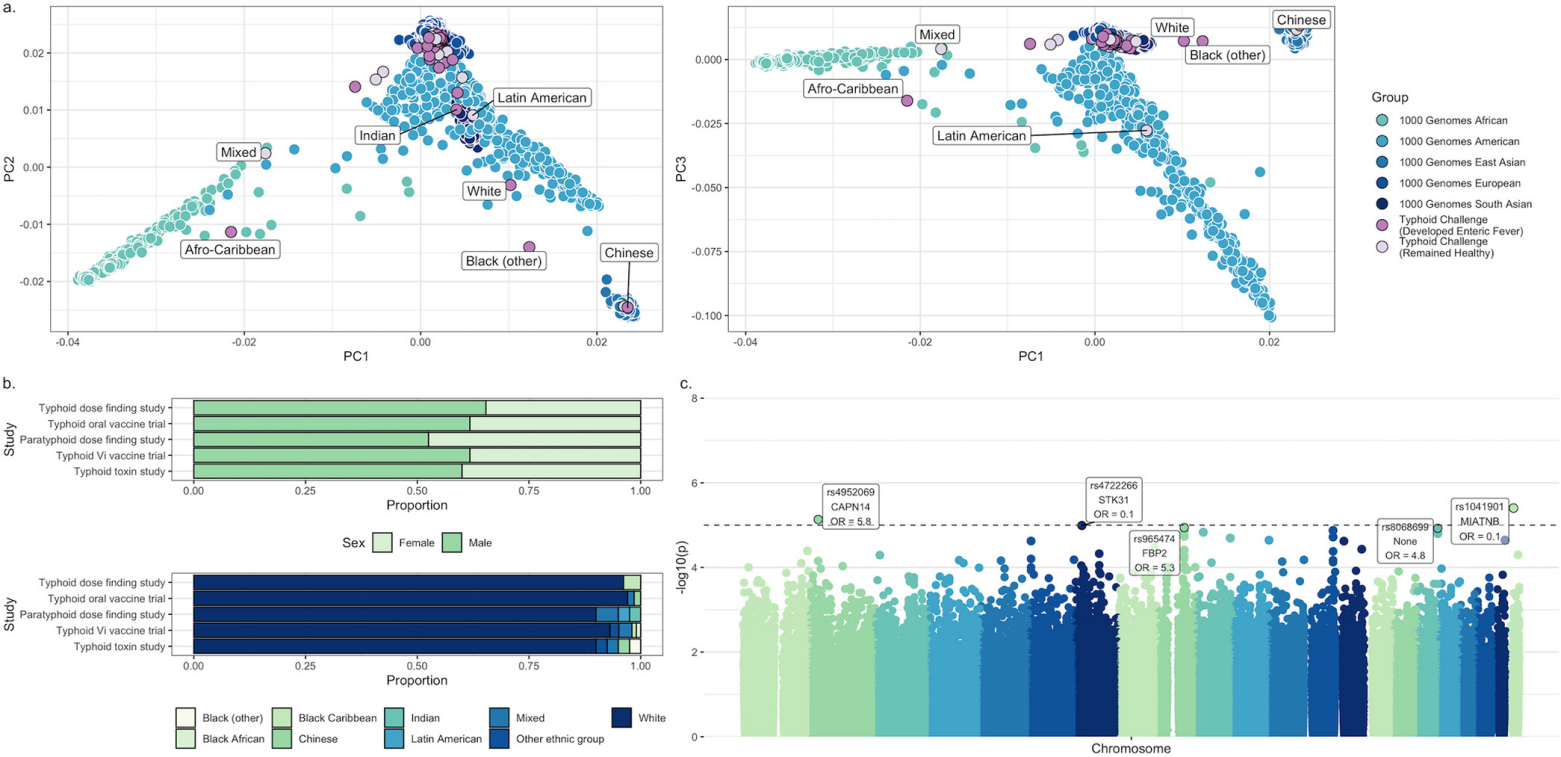
(a) Population structure (principal components 1, 2, and 3) of the enteric fever cohorts within the context of the 1000 Genomes superpopulations. Each point corresponds to one individual, with individuals from the enteric fever cohorts highlighted in purple (light purple = remained healthy following challenge, dark purple = developed enteric fever following challenge) and those from the 1000 Genomes project in blue (differing shades correspond to different superpopulations). Where enteric fever challenge participants do not cluster with the 1000 Genomes European superpopulation, self-reported ethnicity is indicated. (b) Self-reported demographics (sex and ethnicity) of participants included in the GWAS and HLA analyses, by study. (c) Manhattan plot showing the significance [−log_10_(unadjusted *P* value)] of the relationship between each single nucleotide polymorphism (SNP) and development of symptoms or bacteremia following oral S. Typhi or *S.* Paratyphi A challenge, for each chromosome. The dashed line indicates a suggestive *P* value of 10^−5^. The five SNPs with the lowest *P* values are highlighted, with the overlapping gene as identified by SNPnexus indicated as well as the odds ratio (OR).

A genome-wide association analysis was carried out on genotyped participants (101 cases of enteric fever, 68 controls following data cleaning) in order to identify any SNPs associated with development of fever, symptoms, or bacteremia following S. Typhi or *S.* Paratyphi A challenge (Fig. 1). In a principal-component analysis, participants predominantly clustered with the 1000 Genomes European superpopulation (Fig. 2a) and self-reported ethnicity was predominantly white (Fig. 2b). Two SNPs within the genes *CAPN14* and *MIATNB* gave a *P* value below 1 × 10^−5^ (Fig. 2c; see also Fig. S1 and Table S2 in the supplemental material). However, no SNPs reached genome-wide significance, with *P* values exceeding that expected by chance (Fig. S2).

### HLA-B*27:05 is the allele with the strongest association with enteric fever susceptibility.

**FIG 3:**
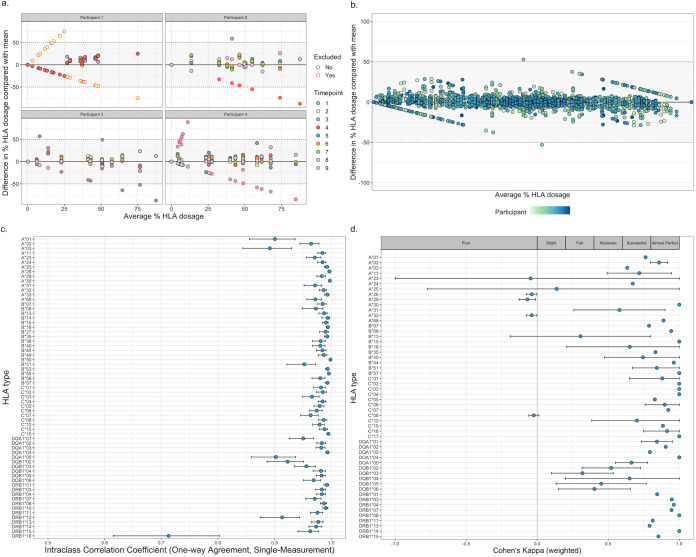
(a) Difference in HLA dosage versus mean HLA dosage for four participants with an outlying time point. For each participant and each HLA type (2-digit resolution), the mean dosage of each HLA type was calculated, as well as the amount by which each time point deviated from the mean. Each point represents an HLA type at a certain time point, color coded by time point. Points which were subsequently excluded due to belonging to an outlying time point are circled in red. The thresholds at which points deviate >50% from the mean are indicated. (b) Difference in HLA dosage versus mean HLA dosage for all participants with multiple time points profiled (*n* = 50) following exclusion of outlying time points. For each participant and each HLA type (2-digit resolution), the mean dosage of each HLA type was calculated, as well as the amount by which each time point deviated from the mean. Each point represents an HLA type in a certain participant at a certain time point, color coded by participant. The thresholds at which points deviate >50% from the mean are indicated. (c) Intraclass correlation coefficients (one way, single measurement) for agreement between HLA type (2-digit resolution) dosages at different time points, as calculated by the R package irrNA. Each point represents the intraclass coefficient for one HLA type, with 95% confidence intervals indicated by error bars. (d) Agreement (weighted Cohen’s kappa) between SNP2HLA and HISAT-genotype for participants HLA typed by both methods (*n* = 71), as calculated by the R package irr. Each point represents the weighted Cohen’s kappa for one HLA type (2-digit resolution), with 95% confidence intervals indicated by error bars. The strength of agreement for each range of kappa, as assigned in the work of Landis and Koch (14), is indicated.

Given that the number of individuals was too small to identify SNPs at the genome-wide level, we then focused on variation within the HLA region. HLA typing was performed either by imputation from genotyping data using SNP2HLA (11) or from raw RNA-sequencing data using HISAT-genotype (12). For 50 participants multiple RNA-sequencing time points were available. To assess the repeatability of HISAT-genotype, the mean dosage, and the amount by which each time point deviated from the mean dosage, was calculated for each participant and HLA type (Fig. S3a) (13). At two-digit resolution, nine comparisons had a difference of >50%, of which eight resulted from one outlying time point in each of four participants (Fig. 3a). These time points were therefore excluded, and differences were recalculated. Ninety-six percent of comparisons where mean dosage of an HLA type exceeded 0% fell within 10% of the mean (Fig. 3b; Fig. S2b). At a two-digit resolution, all HLA types had a one-way intraclass correlation coefficient (ICC) of >0.7 for agreement, and 63/65 had an ICC of >0.9 (Fig. 3c). At a four-digit resolution, 114/126 HLA types had an ICC of >0.7, and 106/126 had an ICC of >0.9 (Fig. S2c).

For participants with multiple time points, the median HLA type dosage was taken. HLA dosages were then rounded to 50%. Seventy-one participants had been HLA typed from both genotyping data, using SNP2HLA, and RNA-sequencing data, using HISAT-genotype. To assess agreement between SNP2HLA and HISAT-genotype, weighted Cohen’s kappa was calculated for each of the HLA types present in these participants. At a two-digit resolution, 46/57 HLA types had a kappa of >0.4 (moderate agreement [14]), 42/57 had a kappa of >0.6 (substantial agreement), and 30/57 had a kappa of >0.8 (almost perfect agreement) (Fig. 3d). At a four-digit resolution, 57/69 HLA types had a kappa of >0.4, 45/69 had a kappa of >0.6, and 27/49 had a kappa of >0.8 (Fig. S2d). HLA types with a weighted Cohen’s kappa of <0.4, at both two- and four-digit resolution, were excluded from subsequent association analyses.

**FIG 4:**
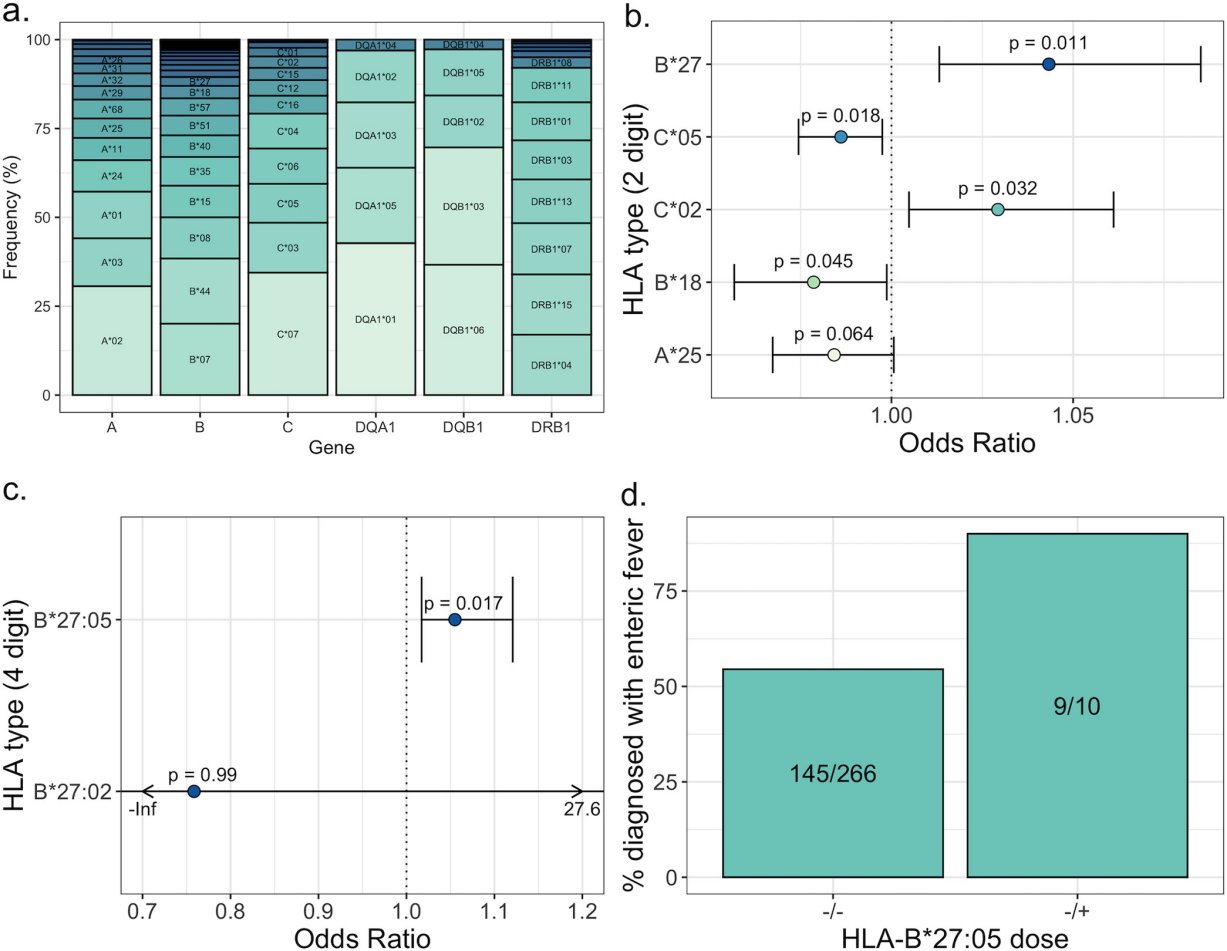
(a) Relative frequency of each HLA type at a resolution of 2 digits for HLA-A, HLA-B, HLA-C, HLA-DQA1, HLA-DQB1, and HLA-DRB1 in the entire combined cohort, including participants from the typhoid dose-finding study, typhoid oral vaccine trial, typhoid Vi vaccine trial, paratyphoid dose-finding study, and typhoid toxin study. (b) Odds ratios (odds ratio of >1 indicates association with susceptibility and of <1 indicates association with resistance) and 95% confidence intervals for the five HLA types most significantly associated with outcome of challenge at a resolution of 2 digits. *P* values are indicated for each. (c) Odds ratios for the two HLA-B*27 subtypes at a resolution of 4 digits with 95% confidence intervals. *P* values are indicated for each. (d) Percentage of participants who were diagnosed with enteric fever following challenge, stratified by the presence or absence of one copy of HLA-B*27:05. The proportion of participants diagnosed is indicated for each group.

The most common HLA-A, -B, -C, -DQA, -DQB1, and -DRB1 allele groups were A*02, B*07, C*07, DQA*01, DQB1*06, and DRB1*04, respectively (Fig. 4a; Table S3). To identify whether any HLA types were associated with enteric fever (diagnosed on the basis of clinical symptoms or positive blood culture), a logistic regression was carried out on HLA types at a 2-digit resolution (Table S4). Two hundred seventy-six participants, of whom 154 were diagnosed with enteric fever, were included in this analysis. The HLA type most associated with susceptibility was HLA-B*27 (*P* = 0.011, odds ratio = 1.04, 95% confidence intervals 1.01 to 1.09) (Fig. 4b). Limiting cases to either those diagnosed on the basis of clinical symptoms only or those diagnosed on the basis of positive blood culture only gave the same result (Fig. S4). While a small odds ratio and a high false-discovery rate (*P* value adjusted for testing 81 HLA types = 0.68) (Fig. S5), this finding was of particular interest as HLA-B*27 has been associated with nontyphoidal Salmonella-induced reactive arthritis and ankylosing spondylitis (15–18). Only the HLA-B*27:05 and B*27:02 subtypes were present in our cohort, and at 4-digit resolution this association was driven by HLA-B*27:05 (Fig. 4c). Of 10 participants heterozygous for HLA-B*27:05, 9 were diagnosed with enteric fever (Fig. 4d).

**FIG 5:**
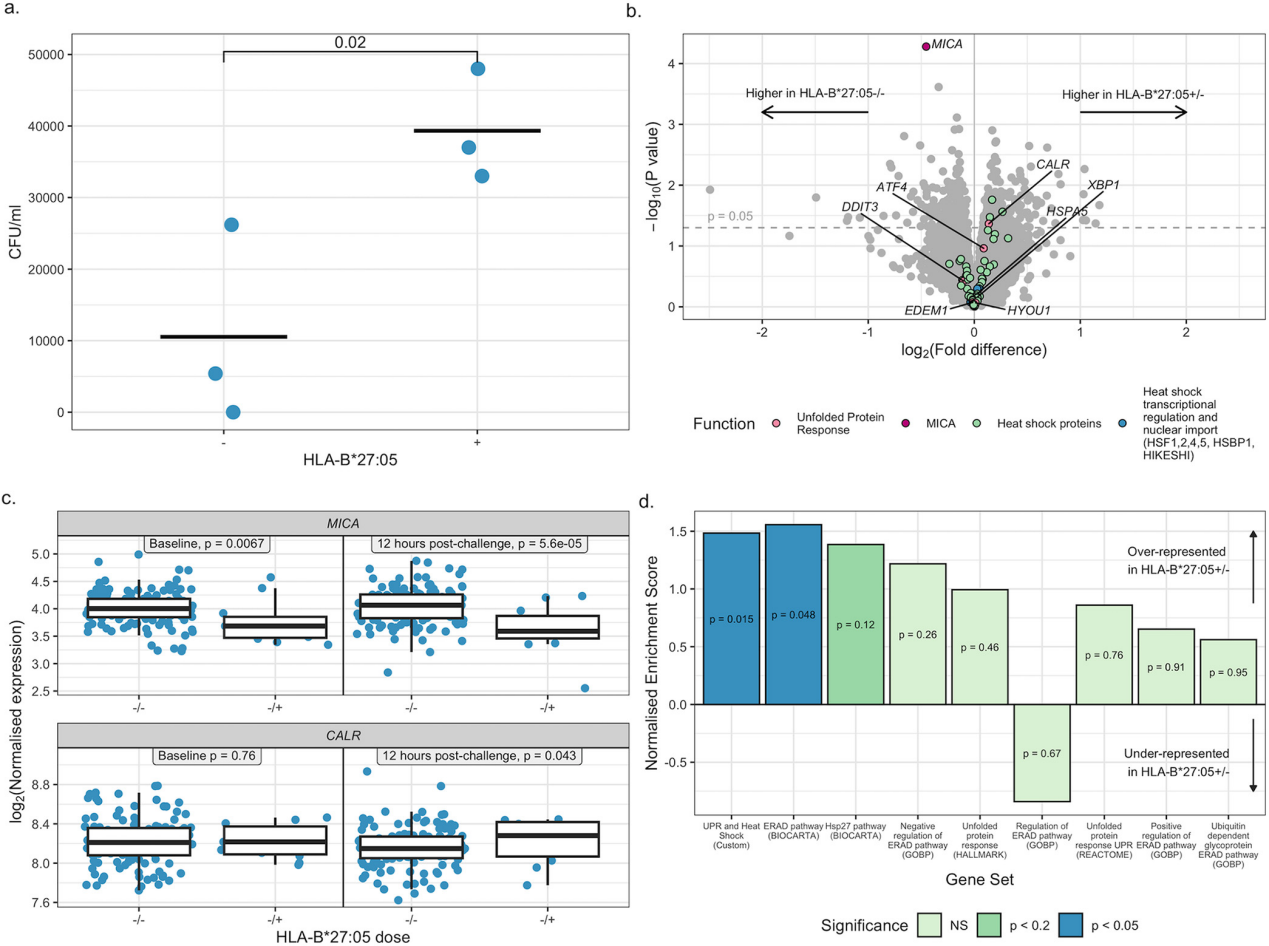
(a) CFU per milliliter recovered from C1R cells infected with S. Typhi Quailes strain, in the presence or absence of HLA-B*27 expression, 24 h postinfection. Parent and HLA-B*27:05^+^ cells were seeded in a 96-well plate at a density of 100,000 cells per well and infected with the S. Typhi Quailes strain at an MOI of 0 or 10 in triplicate. After 1 h gentamicin was added to kill extracellular bacteria. At 24 h postinoculation, cells were lysed using 1% Triton X-100, and lysates were serially diluted and plated onto tryptone soya agar. Colonies were counted following overnight incubation at 37°C. A *P* value for a *t* test is indicated. Points represent replicates within a single experiment. (b) Volcano plot showing the log_2_(fold difference) in gene expression between HLA-B*27:05-positive and -negative participants 12 h postchallenge against the −log_10_(*P* value). A dashed line indicating where *P* equals 0.05 is shown, and genes relating to the unfolded protein response and heat shock proteins are highlighted. Genes more highly expressed in participants who were HLA-B*27:05 positive are shown further to the right, and those more highly expressed in HLA-B*27:05-negative participants are shown further to the left. RNA expression was characterized by RNA sequencing. Data were filtered, normalized, and transformed, and differential expression was then assessed using the limma R package, using participant ID, sequencing pool, vaccination status, challenge strain, and dose as blocking variables. (c) Expression of *MICA* and *CALR* following normalization and transformation using the edgeR and limma packages, in HLA-B*27:05-positive and -negative participants at baseline and 12 h postchallenge. Nominal *P* values before adjustment for multiple testing are indicated. (d) Normalized enrichment scores for gene sets relating to the unfolded protein response (UPR), heat shock, and endoplasmic reticulum-associated protein degradation (ERAD) 12 h following enteric fever challenge in participants heterozygous for HLA-B*27:05 relative to those without HLA-B*27:05. Genes were ranked by *t* statistic, and relevant gene sets were downloaded from the Molecular Signatures Database. A custom gene set based on the genes highlighted in panel b was also included. Gene set enrichment analysis was carried out on the preranked list using GSEA_4.1.0. Bars are color coded and ordered by significance, and nominal *P* values are indicated.

HLA-B*27:05 has previously been found to enhance intracellular growth of S. Typhimurium and Salmonella enterica serovar Enteritidis (19, 20). To investigate whether HLA-B*27:05 may enhance S. Typhi replication, human B-cell lymphoblastoid C1R cells transfected with HLA-B*27:05 were infected with S. Typhi *in vitro* for 24 h. Compared with nontransfected controls, higher numbers of viable bacteria were recovered from HLA-B*27:05^+^ cells (Fig. 5a), suggesting a mechanism independent of antigen presentation. This is consistent with previous literature finding that HLA-B*27:05 lowers the threshold for induction of the unfolded protein response, a pathway that is induced by and enhances intracellular S. Typhimurium infection (19).

To investigate whether differences in the unfolded protein response can be detected in human challenge participants, we explored transcriptional differences between those who did and did not possess a copy of HLA-B*27:05 in the paratyphoid dose finding and Vi vaccine trial studies. We hypothesized that outcome of challenge is dependent on events occurring early after exposure and preceding development of acute disease and therefore focused on 12 h postchallenge, the time point at which dissemination of typhoidal Salmonella is thought to take place in the blood (21, 22). At 12 h post-challenge with S. Typhi or *S*. Paratyphi A, the most significant differentially expressed gene between the two groups was *MICA* (major histocompatibility complex [MHC] class I polypeptide-related sequence A), encoding a ligand for natural killer cell activating receptor NKG2D (Fig. 5b and c). Expression of *MICA* is inhibited by the unfolded protein response (23) and was expressed at lower levels by those with a copy of HLA-B*27:05 (nominal *P* = 0.00005 12 h postchallenge, *P* = 0.0036 adjusting for multiple testing of 67 transcripts hypothesized to be involved in the unfolded protein/heat shock response, and *P* = 0.22 adjusting for multiple testing of 12,194 transcripts in total; nominal *P* = 0.006 at baseline, *P* = 0.44 adjusting for multiple testing of 67 transcripts hypothesized to be involved in the unfolded protein/heat shock response, and *P* = 0.95 adjusting for multiple testing of 12,194 transcripts in total; linear modeling). The gene *CALR* encoding the calcium-binding chaperone calreticulin was more highly expressed in those with HLA-B*27:05 at 12 h postchallenge but not at baseline and not after adjustment for multiple testing (nominal *P* = 0.04 12 h postchallenge, *P* = 0.58 adjusting for multiple testing of 67 transcripts hypothesized to be involved in the unfolded protein/heat shock response, and *P* = 0.62 adjusting for multiple testing of 12,194 transcripts in total; nominal *P* = 0.8 at baseline, *P* = 0.96 adjusting for multiple testing of 67 transcripts hypothesized to be involved in the unfolded protein/heat shock response, and *P* = 0.98 adjusting for multiple testing of 12,194 transcripts in total) (Fig. 5c). The *P* values and log(fold change) for all other transcripts are outlined in Data Set S6 in the supplemental material. Gene set enrichment analysis (24) was then used to assess whether transcripts encoding proteins involved in the unfolded protein response, heat shock response, or endoplasmic reticulum-associated protein degradation were enriched among those with HLA-B*27:05. Eight/nine gene sets had a positive normalized enrichment score, suggesting a trend toward overrepresentation in HLA-B*27:05^+^ participants. A custom gene set containing *CALR*, *ATF4*, *DDIT3*, *HSPA5*, *XBP1*, *EDEM1*, and *HYOU1* as well as 60 genes annotated as relating to the heat shock response was significantly overrepresented in those with HLA-B*27:05 at 12 h postchallenge when ranked by *t*-statistic (*P* = 0.015) (Fig. 5d), as was a gene set relating to endoplasmic reticulum-associated protein degradation (*P* = 0.048). No gene sets were significantly over- or underrepresented at baseline.

## DISCUSSION

This study investigated genetic susceptibility to enteric fever in a human challenge setting. We found HISAT-genotype to be a consistent tool to impute HLA types from RNA-sequencing data, with substantial agreement with SNP2HLA dosages imputed from genotyping data. Of the HLA types, HLA-B*27:05 was most associated with susceptibility to infection (*P* = 0.011). As an MHC class I allele, the primary function of HLA-B*27 is to present antigenic peptides to CD8^+^ cells (25). However, HLA-B*27 has a tendency to misfold, form homodimers, and accumulate in intracellular vesicles, activating endoplasmic reticulum-associated protein degradation (ERAD) and reducing the threshold for activation of the unfolded protein response (26–28). This tendency is hypothesized to relate to its association with ankylosing spondylitis and reactive arthritis following Gram-negative bacterial infection (19, 29). Whereas the subtypes HLA-B*27:05, -:04, and -:02 are associated with ankylosing spondylitis, HLA-B*27:06 and HLA-B*27:09 are not, despite HLA-B*27:05 and 27:09 differing by one amino acid (26). This is due to a destabilizing aspartate residue in the peptide binding pocket of HLA-B*27:05, increasing conformational disorder by repelling other residues (27, 30).

While there is conflicting evidence regarding whether HLA-B*27 modulates cellular invasion by S. Typhimurium and S. Enteritidis (31–33), when infected *in vitro* with S. Typhimurium, both monocyte-like U937 cells and epithelial HeLa cells transfected with HLA-B*27:05 exhibit higher levels of intracellular replication (19, 34). Furthermore, HLA-B*27 enhances intracellular survival of S. Enteritidis in mouse fibroblasts (35). Although the exact mechanism is unknown, the unfolded protein response appears to create a favorable environment for S. Typhimurium, the presence of HLA-B*27:05 increasing its expression of SPI-2 genes (36) and causing it to replicate at the cell periphery (19). Stabilization of HLA-B*27:05 by site-directed mutagenesis or fusion with β2-microglobulin have been found to prevent enhancement of S. Enteritidis and S. Typhimurium replication, respectively (19, 34). Pharmacological induction of endoplasmic reticulum stress by thapsigargin enhances S. Typhimurium replication, while infection with S. Typhimurium stimulates the unfolded protein response by a mechanism dependent on bacterial effector *sifA* (19). Despite belonging to the same species, the pathogenesis and immune response in S. Typhi infection are distinct from those of S. Typhimurium and S. Enteritidis (37). For example, although *sifA* is also present in S. Typhi, its sequence differs from *sifA* in S. Typhimurium (38).

We carried out intracellular growth experiments in C1R cells in order to investigate whether this phenomenon is serovar specific and observed enhanced replication (*P* = 0.02, one-tailed *t* test). C1R cells were used as they have no surface HLA-A/B expression, express large amounts of HLA-B*27, and have previously been used in a number of experiments to explore the contribution of HLA-B*27 to S. Typhimurium- and S. Enteritidis-induced reactive arthritis (32, 33, 39, 40). Furthermore, B cells are *in vivo* targets of S. Typhimurium infection and are highly resistant to Salmonella*-*induced cell death (41, 42). However, our experiment could be improved by quantifying initial invasion, which would allow us to assess how much the bacterial population has grown over 24 h; increasing the number of replicates over multiple experiments; quantifying survival of C1R cells; directly measuring presence of an unfolded protein response; and infecting professional phagocytes, which are thought to be the primary cellular target of S. Typhi. While previous studies have found that control HLA transfectants such as HLA-A*02, B*07, and B*35 do not affect the unfolded protein response or intracellular Salmonella replication (19, 34, 35, 43), inclusion of these could further enhance the validity of this finding. Finally, carrying intracellular growth assays in peripheral blood mononuclear cells from human challenge participants with and without HLA-B*27 would allow us to identify whether this relationship holds in cells with differing genetic backgrounds.

The gene encoding endoplasmic reticulum chaperone calreticulin, *CALR*, was somewhat higher in HLA-B*27:05^+^ human volunteers 12 h following enteric fever challenge but not at baseline (*P* = 0.04 12 h postchallenge, *P* = 0.8 at baseline). Gene set enrichment analysis (24) was then used to assess whether transcripts encoding proteins involved in the unfolded protein response, heat shock response, or ERAD were enriched among those with HLA-B*27:05. Eight/nine gene sets had a positive enrichment score, of which two were significantly overrepresented in those with HLA-B*27:05 at 12 h postchallenge: a custom gene set containing genes annotated as relating to the unfolded protein response and heat shock response (*P* = 0.015) and the BioCarta ERAD pathway gene set (*P* = 0.048). However, no gene sets were overrepresented at baseline. This supports the hypothesis that HLA-B*27:05 reduces the threshold for unfolded protein response activation in infection. At 12 h postchallenge, the most significant differentially expressed gene between the two groups was *MICA*, encoding a ligand for natural killer cell activating receptor NKG2D (*P* = 0.00005, linear modeling). *MICA* is downregulated by the unfolded protein response (23) and was expressed at lower levels in participants with HLA-B*27:05 12 h postchallenge. In viral infections, downregulation of *MICA* prevents recognition by NK cells (44). Polymorphisms in *MICA* have been related to susceptibility to leprosy, which, in common with enteric fever, infects mononuclear phagocytes (45–47). In contrast to *CALR*, *MICA* was also differentially expressed in HLA-B*27:05^+^ participants at baseline (*P* = 0.006, linear modeling), suggesting either that HLA-B*27:05 can induce certain aspects of the unfolded protein response in the absence of infection or that its decreased expression is mediated by a different mechanism.

In the absence of SNPs with very high odds ratios in our cohort, we were underpowered to detect significant SNPs at a genome-wide level. The SNP with the second lowest *P* value (rs4952069, 3.95 × 10^−6^) falls in the intronic region of *CAPN14*, a calcium-dependent cysteine protease regulated by Th2 cytokines interleukin-13 (IL-13) and IL-4 (48). Intronic SNPs may either be linked to a causative coding SNP or themselves affect gene expression through splicing or transcription factor binding (49). *CAPN14* is thought to play a regulatory role in the esophageal epithelium, with overexpression impairing barrier function and SNPs in this locus having been associated with susceptibility to the allergic inflammatory disease eosinophilic esophagitis (50) and middle ear infection (51). While the cellular response to enteric fever infection is Th1 dominated, Th2 cytokines may be modulated by infection, with S. Typhi-specific IL-13 secretion observed in peripheral blood mononuclear cells isolated during typhoid fever convalescence (52) and IL-4 secreted at the apical side of intestinal biopsy specimens infected *in vitro* with S. Typhi (53). Coinfection of mice with both S. Typhimurium and Th2-inducing hookworms impairs clearance of S. Typhimurium, suggesting that polarization toward a Th2 response could be detrimental (54). Therefore, genetic variations predisposing individuals to a more Th2-dominant response to infection could feasibly affect susceptibility to enteric fever.

This is the first genetic study to investigate susceptibility to infection using samples obtained from human challenge volunteers. Furthermore, while HLA-B*27:05 has been linked to nontyphoidal Salmonella infections, this is the first study to find a potential association with enteric fever. However, we were limited by several factors. First, there were cases where the HLA type of a participant was ambiguous, predominantly due to SNP2HLA suggesting several possible HLA types, but also incomplete agreement between SNP2HLA and HISAT-genotype dosages. Second, due to the nature of human challenge studies, our sample size was smaller than conventional GWAS. While notable GWAS with smaller samples than ours have included those associating genetic variants with vitiligo and response to anti-tumor necrosis factor (anti-TNF) treatment, a larger sample would have enabled us to detect associations with smaller effect sizes (55, 56). Only 10 participants were unambiguously identified as HLA-B*27:05^+^, and the odds ratio was small in magnitude. In light of the limited sample size, the association may represent a false positive, and future independent replication is needed. Furthermore, despite having greater control over exposure than case-control studies, the sample was relatively heterogenous in oral dose, strain, and vaccination status. However, given previous evidence of an association with both Salmonella*-*induced reactive arthritis and intracellular S. Typhimurium replication, this intriguing association warrants validation by further studies.

Finally, this study was carried out in a predominantly European cohort not previously exposed to typhoidal Salmonella. While this allowed us to investigate genetic susceptibility without the confounding factor of previous exposure, it is not representative of the population in a setting of endemicity. However, it could have implications for travel medicine: for example, those with a family history of ankylosing spondylitis could be strongly encouraged to undergo typhoid vaccination prior to traveling. Although reactive arthritis following live oral typhoid vaccination is a rare complication (57), parenteral vaccination may be preferable in this case. Furthermore, the HLA-B*27:05 allele is common in South Asia, suggesting that HLA-B*27:05^+^ populations are likely naturally exposed to S. Typhi outside the Oxford experimental model (58–60).

## MATERIALS AND METHODS

### Enteric fever human challenge cohorts.

Five enteric fever human challenge cohorts from studies conducted at the Centre for Clinical Vaccinology and Tropical Medicine (Churchill Hospital, Oxford, UK) were included in this analysis: a typhoid dose-finding study, a paratyphoid dose-finding study, a typhoid oral vaccine study, a typhoid Vi vaccine study, and a study investigating the role of the typhoid toxin, summarized in Fig. 1. All studies were approved by the South Central-Oxford A Research Ethics Committee (10/H0604/53, 11/SC/0302, 14/SC/0004, 14/SC/1427, and 16/SC/0358). All participants provided written informed consent. Following challenge, individuals with fever (sustained oral temperature ≥38°C) or positive blood culture were diagnosed with enteric fever. All challenged participants were treated with ciprofloxacin or azithromycin either at time of diagnosis in diagnosed individuals or after completing the 14-day challenge period if undiagnosed. Peripheral blood samples from participants from five different enteric fever human challenge cohorts were either genotyped or transcriptionally profiled, or in some cases both (Fig. 1). A subset of participants underwent longitudinal transcriptional profiling, with data available from up to nine time points.

### Genotyping.

DNA was extracted from blood clots using a QIA Symphony SP. Briefly, 180 μL of ATL buffer (Qiagen) was added to each clot and then vortexed and incubated overnight at 56°C for lysis. The following day 200 μL of AL buffer (Qiagen) was added to the lysed clot and mixed before transferring 500 μL of the lysate to a 2-mL tube and run on the QIA Symphony using the QIA Symphony DSP DNA Midi kit (Qiagen). The protocol was a customized BC 400 protocol, and DNA was eluted into 100 μL. Samples were quantified using the Qubit and Qubit BR double-stranded DNA (dsDNA) reagents (Invitrogen). Samples from the typhoid dose finding and typhoid oral vaccine trial (total *n* = 96) were genotyped by the Wellcome Trust Centre for Human Genetics using an Illumina OmniExpress-24 v1.0 BeadChip array, while samples from the paratyphoid dose finding study and typhoid toxin study (total *n* = 80) were genotyped by Cambridge Genomic Services using an Illumina OmniExpress-24 v1.3 BeadChip array. Data cleaning for association analysis was carried out in PLINK (61), first on individual and then merged data sets. No participants were excluded on the basis of outlying heterozygosity. Two were excluded due to a mismatch between reported and genotyped sex, and two were excluded after the data sets were merged due to >10% missingness. SNPs with missingness of >10%, minor allele frequency of <10% or Hardy-Weinberg equilibrium test *P* of <0.001 were excluded, both before and after data sets were merged. Pairwise identity by descent identified two samples as identical; the correctly labeled sample was identified by comparison of HISAT-genotype and SNP2HLA HLA types, and the other was excluded. Two participants from different studies were cryptic siblings, of which the participant lacking paired RNA-sequencing data was excluded. Data processing steps are summarized in Fig. 1. Following data cleaning, a total of 479,161 SNPs and 169 participants remained.

For principal-component analysis, 1000 Genomes high-density Omni genotyping data were downloaded from the IGSR FTP site, converted to ped/map format using PLINK, and merged with the enteric fever genotyping data. The overlap between the two data sets was 204,633 SNPs.

Association analysis was carried out using a logistic regression model in PLINK. Challenge dose, vaccination status, challenge strain, age, sex, and 20 principal components (to account for population structure) were included as covariates. The online tool SNPnexus (62) was used to identify genes proximal to SNPs. With the HapMap CEU data set (Utah residents with Northern and Western European ancestry) as a reference, SNP2HLA software (11) was used to impute single nucleotide polymorphisms in the HLA region and identify HLA alleles.

### RNA sequencing.

Whole-blood samples were collected in Tempus Blood RNA tubes. RNA samples from the paratyphoid dose-finding and Vi vaccine trial were poly(A) selected and underwent paired-end sequencing using a HiSeq V4 at the Wellcome Trust Sanger Institute. RNA samples from the typhoid toxin study underwent poly(A) selection and paired-end sequencing at the Beijing Genomics Institute using an Illumina HiSeq4000. Fastq files from the same sample were concatenated. Paired fastq files were aligned to a prebuilt graph reference using HISAT2, followed by extraction of HLA-aligning reads. HLA typing and assembly were then carried out using HISAT-genotype (12).

### HISAT-genotype repeatability and agreement with SNP2HLA.

To assess the repeatability of HISAT-genotype in imputing HLA types, for each HLA type in each of the participants with multiple profiled time points, the difference between HLA type dosage at each time point and the mean dosage was calculated (13). Nine comparisons from four participants gave a difference in dosage of >50%. Of these, eight resulted from a clear outlying time point in each participant. These time points were therefore excluded, and the difference between each time point and the mean was recalculated. For each HLA type, intraclass correlation coefficients (one way, single measurement) were calculated using the irrNA package to assess agreement.

For participants with multiple time points HLA typed by HISAT-genotype, the median HLA type dosage was taken. HLA dosages were then rounded to 50%. For 71 participants both genotyping and RNA-sequencing data were available. To assess the agreement between HLA types imputed by HISAT-genotype and SNP2HLA, weighted Cohen’s kappa was calculated for each HLA type using the irr R package. HLA type dosage was considered to be an ordinal variable (100% >50% >0%). HLA types with a weighted Cohen’s kappa of <0.4, both at 2-digit and at 4-digit resolution, were excluded from association analysis.

### Association between HLA type and outcome.

Dosages were rounded to the nearest 50%, and for participants with multiple time points HLA typed by HISAT-genotype, any time points with outlying dosages (see Fig. S1 in the supplemental material) were excluded and the median for the remaining time points was taken. HLA types where there was no significant correlation (*P* > 0.05) between time points were excluded. For those with both SNP2HLA- and HISAT-genotype-derived HLA types, the mean dosage was then taken. HLA type data from all cohorts were then combined. A logistic regression model was used to assess the relationship between the dosage of each HLA type (0 to 100%) and outcome (1 = diagnosed with enteric fever, 0 = remained undiagnosed). Vaccination status, challenge dose, challenge strain, age, and sex were included as covariates. Statistical tests were carried out in R.

### Intracellular survival of S. Typhi in HLA-B*27:05^+^ cells.

HLA-B*27:05^+^ C1R cells generated using lentiviral constructs were provided by the Bowness Group (63). Transfected control and HLA-B*27:05^+^ cells were seeded in a 96-well plate at a density of 100,000 cells per well. A frozen glycerol stock of 5 × 10^8^ CFU/mL S. Typhi Quailes strain was thawed and washed twice with RPMI 1640 medium. Cells were inoculated at a multiplicity of infection (MOI) of 50 in triplicate. After 1 h, gentamicin was added at a concentration of 200 μg/mL. At 24 h postinoculation cells were washed twice with RPMI and then resuspended in 50 μL 1% Triton X-100. After 2 min, lysates were serially diluted in phosphate-buffered saline and plated onto tryptone soya agar. Colonies were counted following overnight incubation at 37°C. A one-tailed *t* test was used to assess whether the number of colonies was higher in HLA-B*27:05^+^ cells.

### Differences in gene expression in those with HLA-B*27:05.

Prealignment quality control on sequenced samples from the paratyphoid dose-finding study and Vi vaccine trial was carried out using FASTQC. As all files had high Phred scores (>25) across their length, all were aligned to the human genome (GRCh38 Gencode version 26) using STAR-2.6.1c (64). Total reads per sample ranged from 16 to 44 million. Reads per gene were counted using the STAR GeneCounts mode. Principal-component analysis was used for outlier detection, with no samples excluded on this basis. Non-protein-coding and hemoglobin subunit genes were excluded. Count tables were filtered to exclude genes with <1 count per million (cpm) in >31 samples (the number of baseline samples in control participants challenged with S. Typhi) and normalized using weighted trimmed mean of M-value scaling (edgeR). The count matrix was transformed using limma voom and a linear regression model fitted with vaccination status, challenge strain, sequence pool, and dose as covariates and participant identifier (ID) as a blocking variable. At baseline and 12 h postchallenge, differential gene expression analysis between those with and without a copy of HLA-B*27:05 was carried out, filtering to genes with average log_2_(expression) >0.

### Gene set enrichment analysis.

Differences in gene expression between human challenge participants with and without a copy of HLA-B*27:05 were ranked by *t*-statistic at both baseline and 12 h postchallenge. The entire ranked gene list, including nonsignificantly differentially expressed genes, was input into GSEA 4.1.0 software (24). Eight gene sets relevant to the unfolded protein response, heat shock response, or endoplasmic reticulum-associated protein degradation (ERAD) were downloaded from the molecular signatures database (M22013, M2587, M5922, M10294, M15415, M16695, M40515, and M16460). A custom gene set containing genes relating to the unfolded protein and heat shock response was also created (Table S1). An enrichment score reflecting the degree to which these genes were overrepresented at the top of each ranked gene list was calculated. The *P* value of the enrichment score was then calculated by the GSEA 4.1.0 software using an empirical phenotype-based permutation test procedure (24).

### Data availability.

The data supporting the findings of this study are available within the supplemental material, including merged genome-wide association data from all cohorts in bed/bim/fam format (Data Sets S1, S2, and S3) and the associated covariates (Data Set S4), imputed HLA types for each participant (Data Set S5), RNA-sequencing count table (Data Set S6), and the associated metadata (Data Set S7).
