# Supplementary material for: Genetic Susceptibility to Enteric Fever in Experimentally Challenged Human Volunteers
Source: Infect Immun. 2022 Mar 7;90(4):e00389-21. doi: 10.1128/iai.00389-21 (PMC9022534; doi:10.1128/iai.00389-21)
Supplement: Supplemental file 1 — Supplemental material. Download iai.00389-21-s0008.pdf, PDF file, 1.1 MB [file iai.00389-21-s0008.pdf]

Supplementary figures

Figure S1: Seven loci containing the 10 SNPs with the strongest association with outcome of enteric fever challenge. In the top panel, position on the chromosome is shown on the x axis, and magnitude of association ($-\log_{10}$(p value)) is on the y axis. Each point represents one genotyped SNP, with linkage disequilibrium (estimated from the 1000 Genomes project) to the index SNP indicated by colour. The bottom panel shows the location of nearby genes in the UCSC Genome Browser.

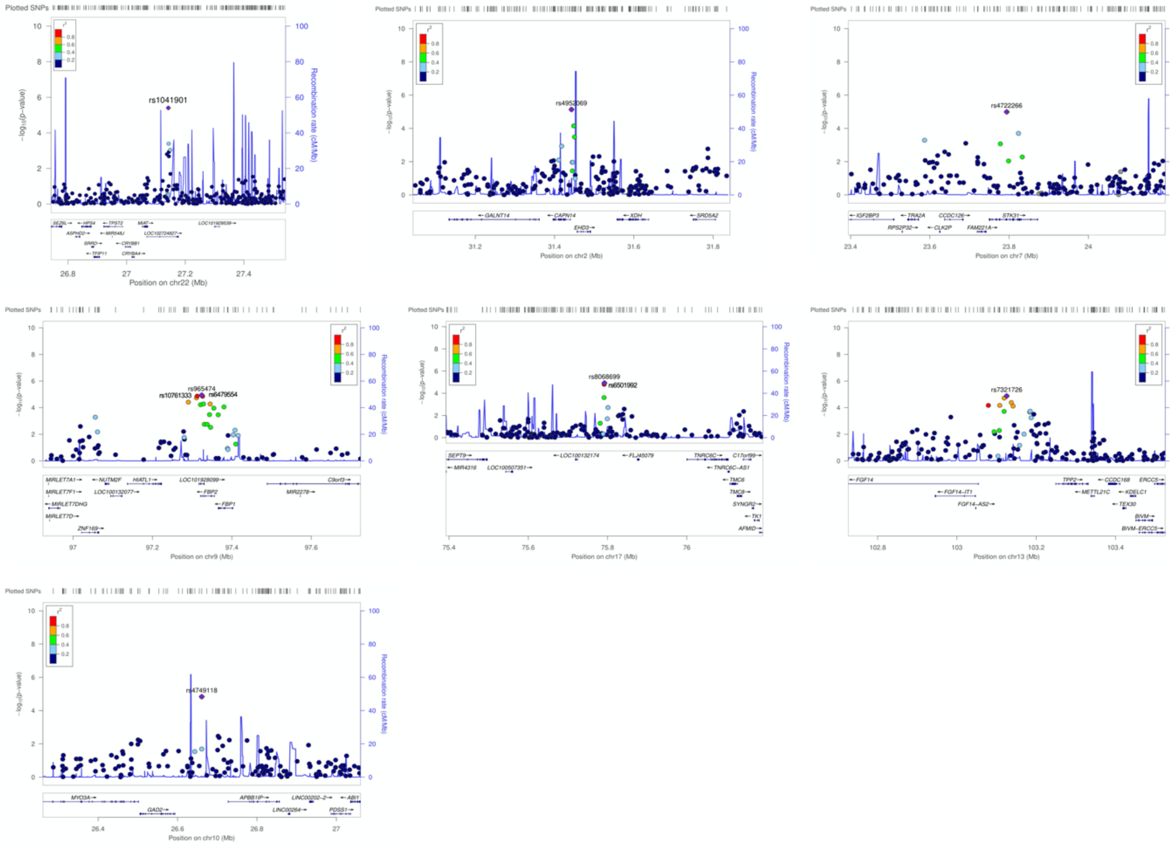

Figure S2: Quantile-Quantile (QQ) plot comparing expected -$\log_{10}$(p values) and observed -$\log_{10}$(p values) for association of SNPs with the development of symptoms or bacteraemia following oral *S.* Typhi or *S.* Paratyphi A challenge. A line of equality is indicated in red.

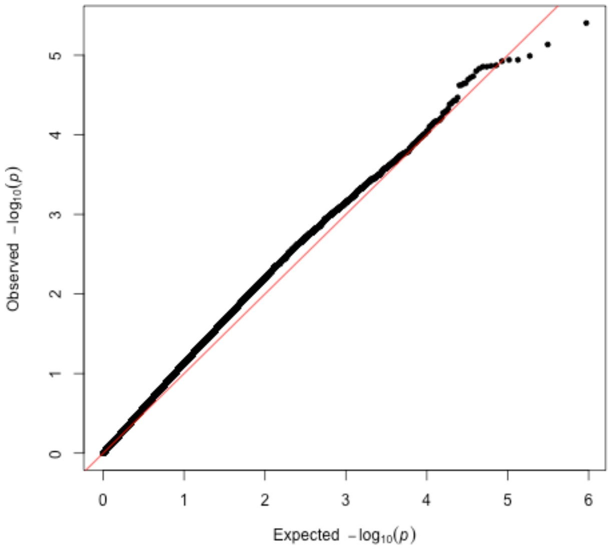

Figure S3: a. Difference in HLA dosage versus mean HLA dosage for all participants with multiple time points profiled (n = 50) prior to exclusion of outlying time points. For each participant and each HLA type (2 digit resolution), the mean dosage of each HLA type was calculated, as well as the amount by which each time point deviated from the mean. Each point represents an HLA type in a certain participant at a certain time point, colour coded by participant. The thresholds at which points deviate > 50% from the mean are indicated.

b. Difference in HLA dosage versus mean HLA dosage for all participants with multiple time points profiled (n = 50) following exclusion of outlying time points, with HLA types at 4 digit resolution. For each participant and each HLA type, the mean dosage of each HLA type was calculated, as well as the amount by which each time point deviated from the mean. Each point represents an HLA type in a certain participant at a certain time point, colour coded by participant. The thresholds at which points deviate > 50% from the mean are indicated.

c. Intra-class correlation coefficients (one-way, single-measurement) for agreement between HLA type (4 digit resolution) dosages at different time points, as calculated by the R package irrNA. Each point represents the intra-class co-efficient for one HLA type, with 95% confidence intervals indicated by error bars.

d. Agreement (Weighted Cohen's Kappa) between SNP2HLA and HISAT-genotype for participants HLA typed by both methods (n = 71), as calculated by the R package irr. Each point represents the Weighted Cohen's Kappa for one HLA type (4 digit resolution), with 95% confidence intervals indicated by error bars. The strength of agreement for each range of Kappa, as assigned in Landis & Koch 1997 [19], is indicated.

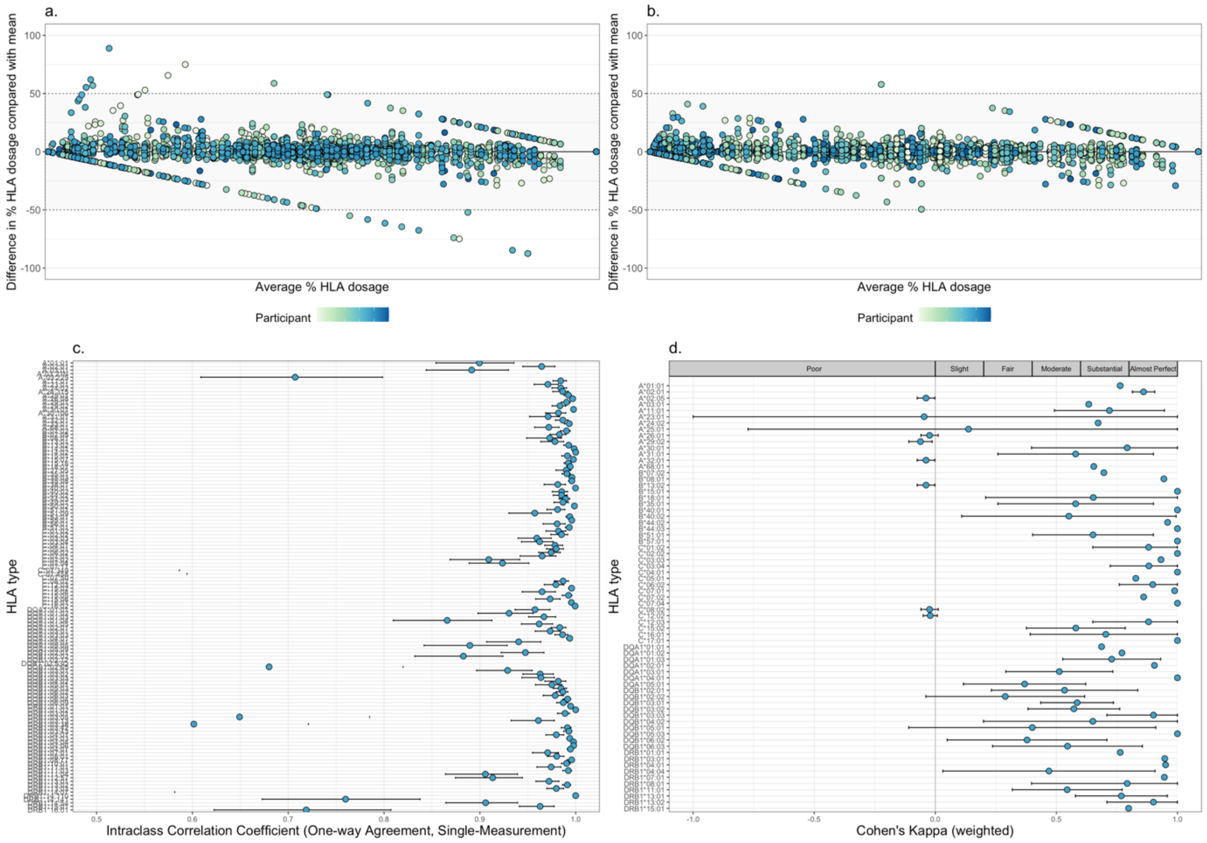

a.
b.
Difference in % HLA dosage compared with mean
Average % HLA dosage
Participant
c.
d.
HLA type
Intraclass Correlation Coefficient (One-way Agreement, Single-Measurement)
Poor
Slight
Fair
Moderate
Substantial
Almost Perfect
Cohen's Kappa (weighted)

Figure S4: Significance of the association between HLA allele groups and enteric fever when limited to diagnosis by clinical symptoms or diagnosis by blood culture only. Data on the mode of diagnosis was available for all studies apart from the typhoid dose-finding study. -$\log_{10}$(p values), representing the significance of the association, are indicated.

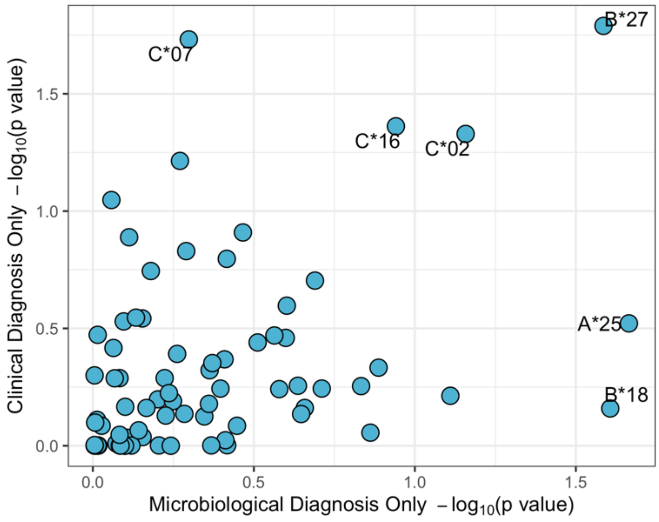

Figure S5: Quantile-Quantile (QQ) plot comparing expected -$\log_{10}$(p values) and observed -$\log_{10}$(p values) for association of 2 digit resolution HLA types with the development of symptoms or bacteraemia following oral *S.* Typhi or *S.* Paratyphi A challenge. A line of equality is indicated in red.

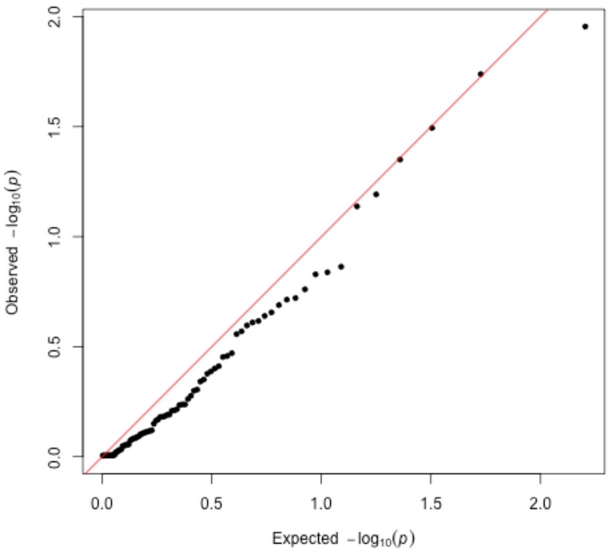

Supplementary tables

Table S1: Genes included in custom unfolded protein response and heat shock response gene set

Family/Function	Genes
Heat shock transcription factors	HSF5, HSF4, HSF2, HSF1
HIKESHI (heat shock protein nuclear import factor)	HIKESHI
HSBP1	HSBP1
Hsp10 sub-family	HSPE1
Hsp110 sub-family	HSPH1
Hsp40 sub-family	DNAJC4, DNAJB1, DNAJC6, DNAJC24, DNAJB5, DNAJC3, DNAJC19, DNAJA4, DNAJC9, DNAJB4, DNAJC15, DNAJA3, DNAJC17, DNAJC25, DNAJC30, DNAJC11, DNAJB6, DNAJC18, DNAJC2, DNAJC5, DNAJC1, DNAJC27, DNAJB11, DNAJC14, DNAJB9, DNAJC8, DNAJC7, DNAJC10, DNAJC16, DNAJA1, DNAJA2, DNAJC21, DNAJB14, DNAJB2, DNAJC13, DNAJB12
Hsp60 sub-family	HSPD1
Hsp70 sub-family	HSPA8, HSPA1B, HSPA4, HSPA14, HSPA6, HSPA1L, HSPA13, HSPA5, HSPA1A, HSPA9
Hsp90 sub-family	HSP90AA1, HSP90B1, HSP90AB1
Small HSP sub-family	HSPB1, HSPB11
Unfolded Protein Response	CALR, ATF4, HSBP1L1, DDIT3, XBP1, EDEM1, HYOU1

Table S2: SNPs with the lowest p values and proximal genes. Positive log_{10}(Odds-ratio)s suggest an association with susceptibility to enteric fever, and negative log_{10}(Odds-ratio)s with resistance to enteric fever. SNPnexus was used to identify proximal genes, and Entrez gene summaries to identify gene function.

SNP	Chromosome	p value	MAF	log_{10}(Odds Ratio)	Closest gene	Gene position relative to SNP	Function
rs1041901	22	3.95E-06	0.21	-0.88	MIATNB	Overlap	lncRNA
rs4952069	2	7.34E-06	0.28	0.76	CAPN14	Overlap	Calcium-activated cysteine protease
rs4722266	7	1.02E-05	0.13	-0.99	STK31	Overlap	Serine-threonine kinase
rs965474	9	1.15E-05	0.38	0.73	FBP2	Overlap	Gluconeogenesis enzyme
rs8068699	17	1.19E-05	0.40	0.68	RP11-316M20.1	Upstream	lncRNA
rs7321726	13	1.34E-05	0.28	0.82	RP11-811P12.3	Upstream	lncRNA
rs10761333	9	1.36E-05	0.37	0.75	PCAT7	Downstream	lncRNA
rs6479554	9	1.40E-05	0.40	0.68	FBP2	Overlap	Gluconeogenesis enzyme
rs4749118	10	1.47E-05	0.18	1.08	APBB1IP	Downstream	Regulates integrins and complement-dependent phagocytosis
rs6501992	17	1.59E-05	0.41	0.66	RP11-316M20.1	Upstream	lncRNA

Table S3: HLA type frequencies in the entire combined cohort, at 2 digit allele group and 4 digit allele resolution.

HLA type (2 digit)	Frequency (%)	Frequency (4 digit resolution subtypes)
A*02	30.6	A*02:01 28.9%, A*02:05 0.9%, A*02:06 0.6%, A*02:489 0%, A*02:121 0%
A*03	13.5	A*03:01 13%, A*03:02 0.2%, A*03:225 0%, A*03:21N 0%, A*03:19 0%
A*01	13.1	A*01:01 12.8%, A*01:04N 0%
A*24	8.9	A*24:02 8.9%, A*24:03 0.6%, A*24:289 0%, A*24:315 0%
A*11	6.2	A*11:01 6.2%
A*25	5.5	A*25:01 5.5%
A*68	5.3	A*68:01 5.2%, A*68:02 0.2%
A*29	3.8	A*29:02 3.7%, A*29:01 0.2%
A*32	3.6	A*32:01 3.6%
A*31	2.7	A*31:01 2.8%, A*31:12 0%, A*31:97 0%
A*26	2.1	A*26:01 1.8%, A*26:08 0.1%, A*26:118 0%
A*30	2	A*30:01 1.4%, A*30:02 0.6%, A*30:04 0.1%, A*30:106 0%
A*23	1.4	A*23:01 1.4%
A*33	1	A*33:01 0.8%, A*33:03 0.2%
A*74	0.2	A*74:01 0.2%
A*66	0.1	A*66:01 0.1%
B*07	20.1	B*07:02 19.4%, B*07:06 0.2%, B*07:05 0.2%, B*07:40 0%
B*44	18.3	B*44:02 10.6%, B*44:03 7.9%, B*44:05 0.1%, B*44:04 0%, B*44:09 0%, B*44:152 0%, B*44:19N 0%
B*08	11.6	B*08:01 11.5%
B*15	8.9	B*15:01 7.9%, B*15:10 0.4%, B*15:07 0.2%, B*15:17 0.2%, B*15:18 0.2%
B*35	8.1	B*35:01 6.2%, B*35:03 0.9%, B*35:08 0.5%, B*35:02 0.3%, B*35:17 0.1%
B*40	6.1	B*40:01 5.1%, B*40:02 1.1%
B*51	5.5	B*51:01 5.1%, B*51:09 0.2%, B*51:08 0.1%
B*57	4.9	B*57:01 4.9%
B*18	3.5	B*18:01 3.6%
B*27	2.5	B*27:05 2.2%, B*27:02 0.2%
B*14	1.8	B*14:02 1.6%, B*14:01 0.3%
B*39	1.6	B*39:06 1.4%, B*39:01 0.2%
B*13	1.4	B*13:02 1.3%, B*13:01 0.1%
B*49	1.1	B*49:01 1.1%
B*38	1	B*38:01 1%
B*52	0.8	B*52:01 0.8%
B*55	0.5	B*55:01 0.5%
B*56	0.5	B*56:01 0.5%
B*42	0.4	B*42:01 0.4%

B*37	0.3	B*37:01 0.3%
B*45	0.3	B*45:01 0.3%
B*47	0.2	B*47:01 0.2%
B*50	0.2	B*50:01 0.1%, B*50:02 0.1%
B*53	0.2	B*53:01 0.2%
B*58	0.2	B*58:01 0.2%
B*41	0.1	B*41:02 0.1%, B*41:01 0%
B*81	0.1	B*81:01 0.1%
B*78	0	B*78:02 0%
C*07	34.4	C*07:01 15.8%, C*07:02 15.3%, C*07:04 1.3%, C*07:458 0.9%, C*07:50 0.4%, C*07:11 0.2%, C*07:19 0%, C*07:349 0%, C*07:57 0%
C*03	14.1	C*03:04 8%, C*03:03 6%, C*03:02 0.2%
C*05	10.9	C*05:01 10.9%, C*05:05 0%, C*05:07N 0%, C*05:09 0%, C*05:109 0%, C*05:111 0%, C*05:113N 0%
C*06	10	C*06:02 10.1%, C*06:06 0%
C*04	9.8	C*04:01 9.6%, C*04:09N 0.5%, C*04:30 0%
C*16	5.1	C*16:01 4.6%, C*16:02 0.4%, C*16:04 0.2%
C*12	4.4	C*12:03 3.5%, C*12:02 1%, C*12:113 0%
C*15	3.5	C*15:02 2.9%, C*15:05 0.4%, C*15:04 0.1%, C*15:06 0%
C*02	3.2	C*02:02 3.3%
C*01	2.4	C*01:02 2.3%
C*08	1.6	C*08:02 1.6%
C*17	0.4	C*17:01 0.4%, C*17:03 0%
C*14	0.3	C*14:02 0.3%
C*18	0.1	C*18:01 0.1%
DQA1*01	42.8	DQA1*01:02 22.1%, DQA1*01:01 11.1%, DQA1*01:03 8.4%, DQA1*01:04 1.1%, DQA1*01:05 0.5%, DQA1*01:11 0%
DQA1*05	21.2	DQA1*05:01 15.5%, DQA1*05:05 5%, DQA1*05:09 0%, DQA1*05:10 0%
DQA1*03	18.4	DQA1*03:01 14.3%, DQA1*03:03 3.6%, DQA1*03:02 0.5%
DQA1*02	14.6	DQA1*02:01 14.9%
DQA1*04	3	DQA1*04:01 3.1%
DQB1*06	36.6	DQB1*06:02 22.6%, DQB1*06:03 11.2%, DQB1*06:04 2%, DQB1*06:09 0.6%, DQB1*06:01 0.5%
DQB1*03	33	DQB1*03:01 18.7%, DQB1*03:02 8.9%, DQB1*03:03 4.6%, DQB1*03:19 0.6%, DQB1*03:04 0.2%, DQB1*03:05 0%, DQB1*03:10 0%, DQB1*03:12 0%, DQB1*03:191 0%
DQB1*02	14.6	DQB1*02:02 7.6%, DQB1*02:01 6.7%, DQB1*02:12 0%, DQB1*02:62 0%, DQB1*02:65 0%, DQB1*02:53Q 0%
DQB1*05	13	DQB1*05:01 10%, DQB1*05:03 1.5%, DQB1*05:02 1.3%, DQB1*05:52 0.2%, DQB1*05:04 0%
DQB1*04	2.7	DQB1*04:02 2.8%
DRB1*04	17	DRB1*04:01 10%, DRB1*04:04 4.3%, DRB1*04:03 0.9%, DRB1*04:05 0.5%, DRB1*04:07 0.4%, DRB1*04:02 0.4%, DRB1*04:08 0.2%, DRB1*04:06 0.1%, DRB1*04:110 0%
DRB1*15	17	DRB1*15:01 15.6%, DRB1*15:02 0.6%, DRB1*15:03 0.4%, DRB1*15:96 0%, DRB1*15:07 0%, DRB1*15:66 0%

DRB1*07	14.4	DRB1*07:01 14.6%, DRB1*07:71 0%
DRB1*13	12.3	DRB1*13:01 7.7%, DRB1*13:02 4.4%, DRB1*13:03 0.7%
DRB1*03	11	DRB1*03:01 10.8%, DRB1*03:02 0.2%, DRB1*03:38 0%, DRB1*03:05 0%, DRB1*03:42 0%, DRB1*03:43 0%, DRB1*03:130 0%, DRB1*03:24 0%, DRB1*03:13 0%, DRB1*03:27 0%
DRB1*01	10.7	DRB1*01:01 9.3%, DRB1*01:02 1%, DRB1*01:03 0.4%
DRB1*11	9.7	DRB1*11:01 7.4%, DRB1*11:04 1.6%, DRB1*11:03 0.5%, DRB1*11:02 0.4%
DRB1*08	3	DRB1*08:01 2.6%, DRB1*08:77 0.2%, DRB1*08:04 0.2%, DRB1*08:02 0.1%, DRB1*08:63 0%
DRB1*14	1.8	DRB1*14:01 0.8%, DRB1*14:54 0.5%, DRB1*14:04 0.2%, DRB1*14:141 0%, DRB1*14:103 0%, DRB1*14:110 0%, DRB1*14:07 0%
DRB1*12	1.1	DRB1*12:01 1.1%, DRB1*12:57 0%, DRB1*12:06 0%
DRB1*16	1.1	DRB1*16:01 1.1%, DRB1*16:08 0%, DRB1*16:09 0%
DRB1*10	0.6	DRB1*10:01 0.6%, DRB1*10:02 0%, DRB1*10:12 0%, DRB1*10:14 0%, DRB1*10:17 0%
DRB1*09	0.4	DRB1*09:01 0.4%, DRB1*09:23 0%

Table S4: Odds ratios (odds ratio >1 indicates association with susceptibility and <1 with resistance), 95% confidence intervals, nominal p values and false discovery rate adjusted p values for the all 2 digit resolution HLA types tested for association with outcome of challenge.

HLA type	Odds ratio	Upper 95% Interval	Lower 95% Interval	Nominal p value	Number hetero-zygotes	Number homo-zygotes	FDR adjusted p value
B*27	1.04	1.09	1.01	0.011	12	0	0.731
C*05	0.99	1.00	0.97	0.018	48	5	0.731
C*02	1.03	1.06	1.00	0.032	18	0	0.855
B*18	0.98	1.00	0.96	0.045	15	1	0.893
A*25	0.98	1.00	0.97	0.064	23	1	0.972
C*16	1.02	1.04	1.00	0.073	28	0	0.972
C*01	1.02	1.05	1.00	0.137	11	1	0.989
DQB1*05	1.01	1.02	1.00	0.145	47	9	0.989
C*15	1.02	1.04	1.00	0.148	17	1	0.989
B*39	0.98	1.01	0.94	0.174	7	0	0.989

A*31	0.99	1.01	0.96	0.190	14	0	0.989
A*33	1.03	1.08	0.99	0.193	5	0	0.989
C*07	1.00	1.00	0.99	0.205	115	38	0.989
A*01	0.99	1.00	0.98	0.221	64	6	0.989
A*23	0.98	1.01	0.95	0.229	6	0	0.989
A*29	1.01	1.04	0.99	0.242	18	0	0.989
A*11	0.99	1.01	0.98	0.246	33	1	0.989
B*44	0.99	1.00	0.98	0.253	76	9	0.989
B*49	0.97	1.02	0.92	0.269	4	0	0.989
B*15	1.01	1.02	0.99	0.277	44	1	0.989
DRB1*10	1.03	1.10	0.97	0.339	2	0	0.989
B*35	0.99	1.01	0.98	0.349	37	1	0.989
A*68	1.01	1.02	0.99	0.353	27	0	0.989
C*03	1.00	1.02	0.99	0.388	61	9	0.989
B*51	1.01	1.03	0.99	0.397	25	1	0.989
B*08	1.00	1.01	0.98	0.410	53	3	0.989
DRB1*14	1.01	1.05	0.98	0.420	10	0	0.989
C*08	0.99	1.02	0.95	0.446	7	0	0.989
DRB1*09	0.98	1.04	0.91	0.456	2	0	0.989
C*12	0.99	1.01	0.98	0.496	23	0	0.989
C*06	1.00	1.02	0.99	0.502	53	1	0.989
DRB1*08	1.01	1.03	0.99	0.529	15	1	0.989
DQA1*03	1.00	1.01	0.99	0.547	67	12	0.989
DQB1*06	1.00	1.00	0.99	0.579	77	45	0.989
DRB1*11	1.00	1.01	0.98	0.579	46	3	0.989
A*24	1.00	1.01	0.98	0.582	43	2	0.989
DRB1*04	1.00	1.01	0.99	0.609	76	10	0.989
C*04	1.00	1.01	0.99	0.617	45	5	0.989
DRB1*01	1.00	1.01	0.99	0.619	48	6	0.989

B*57	1.00	1.02	0.99	0.645	23	1	0.989
A*32	1.00	1.02	0.98	0.646	19	0	0.989
B*14	0.99	1.02	0.96	0.656	8	0	0.989
DRB1*13	1.00	1.01	0.99	0.658	60	3	0.989
DRB1*16	1.01	1.05	0.97	0.660	5	0	0.989
B*45	0.98	1.06	0.87	0.678	1	0	0.989
DRB1*15	1.00	1.01	0.99	0.709	79	8	0.989
B*13	1.01	1.05	0.97	0.759	5	0	0.989
B*52	1.01	1.05	0.96	0.765	3	0	0.989
DQA1*01	1.00	1.01	0.99	0.768	143	47	0.989
DQA1*02	1.00	1.01	0.99	0.775	67	6	0.989
B*40	1.00	1.02	0.99	0.778	22	4	0.989
DQB1*02	1.00	1.01	0.99	0.788	63	6	0.989
DQA1*04	1.00	1.02	0.98	0.790	15	1	0.989
B*38	1.01	1.06	0.96	0.815	3	0	0.989
DQA1*05	1.00	1.01	0.99	0.824	85	13	0.989
B*07	1.00	1.01	0.99	0.826	81	10	0.989
B*56	0.99	1.06	0.93	0.835	2	0	0.989
DQB1*03	1.00	1.01	0.99	0.844	98	35	0.989
DRB1*07	1.00	1.01	0.99	0.880	66	7	0.989
A*02	1.00	1.01	0.99	0.881	107	35	0.989
DRB1*03	1.00	1.01	0.99	0.883	56	3	0.989
A*03	1.00	1.01	0.99	0.894	68	4	0.989
B*42	1.00	1.07	0.93	0.895	2	0	0.989
C*14	1.00	1.09	0.89	0.927	1	0	0.989
DQB1*04	1.00	1.02	0.98	0.933	12	1	0.989
B*37	1.00	1.08	0.88	0.937	1	0	0.989
A*26	1.00	1.03	0.97	0.955	10	0	0.989
DRB1*12	1.40		0.27	0.984	6	0	0.989

B*58	1.35		0.04	0.986	1	0	0.989
B*47	1.35		0.04	0.986	1	0	0.989
A*74	0.76	27.74		0.988	1	0	0.989
C*17	1.34		0.06	0.988	2	0	0.989
A*30	1.00	1.02	0.98	0.989	10	1	0.989
